# The PE16 (Rv1430) of *Mycobacterium tuberculosis* Is an Esterase Belonging to Serine Hydrolase Superfamily of Proteins

**DOI:** 10.1371/journal.pone.0055320

**Published:** 2013-02-01

**Authors:** Rafiya Sultana, Mani Harika Vemula, Sharmishta Banerjee, Lalitha Guruprasad

**Affiliations:** 1 School of Chemistry, University of Hyderabad, Hyderabad, India; 2 Department of Biochemistry, School of Life Sciences, University of Hyderabad, Hyderabad, India; University of Padova, Medical School, Italy

## Abstract

The PE and PPE multigene families, first discovered during the sequencing of *M. tuberculosis* H37Rv genome are responsible for antigenic variation and have been shown to induce increased humoral and cell mediated immune response in the host. Using the bioinformatics tools, we had earlier reported that the 225 amino acid residue PE-PPE domain (Pfam: PF08237) common to some PE and PPE proteins has a “serine α/β hydrolase” fold and conserved Ser, Asp and His catalytic triad characteristic of lipase, esterase and cutinase activities. In order to prove experimentally that PE-PPE domain is indeed a serine hydrolase, we have cloned the full-length Rv1430 and its PE-PPE domain into pET-28a vector, expressed the proteins in *E. coli* and purified to homogeneity. The activity assays of both purified proteins were carried out using *p*-nitrophenyl esters of aliphatic carboxylic acids with varying chain length (C2–C16) to study the substrate specificity. To characterize the active site of the PE-PPE domain, we mutated the Ser199 to Ala. The activity of the protein in the presence of serine protease inhibitor- PMSF and the mutant protein were measured. Our results reveal that Rv1430 and its PE-PPE domain possess esterase activity and hydrolyse short to medium chain fatty acid esters with the highest specific activity for pNPC6 at 37°C, 38°C and pH 7.0, 8.0. The details of this work and the observed results are reported in this manuscript.

## Introduction

The complete genome of the *Mycobacterium tuberculosis* H37Rv strain comprises about 4000 genes, of which 250 genes are involved in fatty acid metabolism [1]. The cell wall structure of *M. tuberculosis* deserves special attention because it is unique among prokaryotes and is a major determinant for virulence of the bacterium. The mycobacterial cell wall contains complex peptidoglycan and lipids [2]. Apart from the expected richness of genes involved in fatty acid metabolism, two novel gene families, PE and PPE were identified in H37Rv genome encompassing 10% of coding regions [1]. These two large unrelated families of acidic, glycine-rich proteins are often based on multiple copies of the polymorphic GC-rich repetitive sequences (PGRSs), and major polymorphic tandem repeats (MPTRs). The names PE and PPE are derived from the motifs Pro–Glu (PE) and Pro–Pro–Glu (PPE) often found at the N terminus. The PE and PPE proteins have the characteristic 110 amino acid and 180 amino acid conserved domains towards the N-terminus, respectively [1], [3].

Since their revelation, experiments have been conducted that assigned several physiological roles to different ORFs belonging to these novel families as mentioned below. Transcriptomics revealed that these proteins are expressed under different conditions with 128/169 PE and PPE genes differentially regulated [4]. It is reported that some PE proteins (Rv0746, Rv1759c, Rv1818c) play a role in immune evasion and antigenic variation [1], [5]–[7], Some members of the PE and PPE families (Rv1818c, Rv1917c, Rv3873) are associated with the cell wall [2], [8]. The PE family protein, Rv1818c influences the interactions of mycobacteria with macrophages [9]. The PPE protein, Rv2430c induces a strong B-cell response [10] and some members of the PE and PPE families (Rv1787, Rv2430c, Rv3018c) are associated with virulence [10]–[12]. Some of the PE/PPE members exist as gene pairs that are co-regulated, co-expressed and interact functionally [13]. Several PE/PPE genes were found to be upregulated only during macrophage infection and in host granulomas supporting their role in pathogenesis of mycobacteria [4]. Despite these efforts, several ORFs in PE/PPE gene clusters are largely unannotated with regard to their biochemical activity with the exception of Rv3097c (LipYtub). The C-terminal domain of Rv3097c shares homology with the hormone-sensitive lipase family characterized by the conserved GDSAG active-site motif and was shown to hydrolyze extracellular lipids [14]–[17]. Combined with the facts that these are multigene families with a high potential for functional redundancy as well as diversity, are unique to mycobacteria, have evolutionarily expanded preferentially in pathogenic mycobacteria [18] and are absent in human host, make them ideal for diagnosis and drug targeting for the design of new anti-tuberculosis drugs.

Nearly 50% of these proteins comprise only the characteristic N-terminal conserved domain, while other members comprise C-terminal extensions. Based on the composition of the C-terminal extensions, these were further classified into various subfamilies by Cole et al., 1998. The PPE family proteins comprise (i) the NxGxGNxG, major polymorphic tandem repeats (MPTR) sequence motif, (ii) proteins with a conserved GxxSVPxxW motif around position 350 along the sequence and (iii) other ‘unrelated’ proteins. The PE family proteins are classified based on phylogenetic analyses, the largest subfamily comprises polymorphic GC-rich repetitive sequence class (PGRS) characterized by a high glycine content due to multiple tandem repeats of the Gly-Gly-X type. The other PE subfamilies are known to share very little C-terminal sequence similarity [1]–[3]. In our previous studies, we had shown that some PE, PPE and hypothetical proteins in mycobacterial species have a 225 amino acid residue conserved domain towards the C-terminus [3] that was termed as the PE-PPE domain (Pfam ID: PF08237). In *M. tuberculosis* H37Rv strain 10 proteins (Rv0151c, Rv0152c, Rv0159c, Rv0160c, Rv1430, Rv1800, Rv2608, Rv3539, Rv1184c and Rv3822) comprise the PE-PPE domain. Using the fold prediction methods, we identified that the PE-PPE domain has a “serine α/β hydrolase” structure with the pentapeptide sequence motif GxSxG/S and conserved Ser, Asp and His catalytic triad characteristic of lipase, esterase and cutinase activities [19]. In order to prove that the PE-PPE domain is indeed a hydrolase, we have cloned the full-length Rv1430 and its PE-PPE domain region into pET-28a vector, overexpressed the protein in *E. coli*, purified the protein to homogeneity and carried out biochemical assays to study their enzyme activity. The kinetic parameters of substrate hydrolysis were measured for Rv1430 and its PE-PPE domain. The active site Ser199 was mutated to Ala in the PE-PPE domain to validate its participation in the enzyme activity. The circular dichroism spectra of Rv1430 and its PE-PPE domain were recorded to observe the folding of the proteins. The dependence of enzyme activity on the variation with temperature, pH and ionic strength were also measured. In this work we provide the experimental validation of our hypothesis obtained from computational analysis.

## Materials and Methods

### Reagents Used

We obtained the H37Rv genomic DNA from Blue Peter Research Centre, LEPRA, Hyderabad, India, that was gifted by Colorado State University (Fort Collins, CO, USA). The Jumpstart accutaq DNA polymerase was purchased from Sigma chemicals and the pET-28a vector was purchased from Novagen. The *E. coli* DH5α cells used for plasmid preparations during cloning and the *E. coli* BL21 cells used for protein expression were grown in Luria-Bertani (LB) broth or on LB agar. The isopropyl β-D -1-thiogalactopyranoside (IPTG) and kanamycin were purchased from HiMedia and supplemented as necessary. The substrates, *p*-nitrophenyl-acetate (pNPC2), *p*-nitrophenyl-butyrate (pNPC4), *p*-nitrophenyl-caprylate (pNPC8), *p*-nitrophenyl-caprate (pNPC10), *p*-nitrophenyl-laurate (pNPC12), *p*-nitrophenyl-myristate (pNPC14) and *p*-nitrophenyl-palmitate (pNPC16), Triton X-100, Tween-20, gum arabic and phenylmethylsulfonyl fluoride (PMSF) were purchased from Sigma chemicals. The cobalt metal affinity resin was purchased from Clontech and the substrate, *p*-nitrophenyl-caproate (pNPC6) was purchased from TCI chemicals, India.

### Cloning, Expression and Purification of Recombinant Rv1430 and its PE-PPE Domain

The full-length PE16 gene; Rv1430 (1601 bp) and its PE-PPE domain region from 430–1107 base pairs (678 bp) of *M. tuberculosis* were amplified by PCR from H37Rv genomic DNA using Taq DNA polymerase (proof-reading thermostable DNA polymerase). The forward primer 5′ATGTCGTTCGTTTTCGCGGTGCCA3′, the reverse primer 5′GGGGGCCTATACCGGAAAATCCTG3′ were used for the Rv1430, and the forward primer 5′CACTACTTGGGCTATGCGTTTTCC3′ and the reverse primer 5′TCAGTCATAACCCAATTCGATGATCGC3′ were used for the PE-PPE domain, with *Bam*HI and *Hin*dIII restriction sites. The PCR products were directly cloned into the expression vector pET-28a with hexa histidine tag. The recombinant plasmids were transformed into the *E. coli* DH5α cells. The presence of the inserts was confirmed by *Bam*HI and *Hin*dIII restriction enzyme digestion and DNA sequencing.

The pET-28a vector carrying Rv1430 gene was transformed into *E.coli* Rosetta gami and the pET-28a vector carrying PE-PPE domain region was transformed into the *E. coli* BL21 (DE3) CodonPlus-RIL for optimal expression of proteins. The *E. coli* Rosetta gami and *E. coli* BL21 (DE3) CodonPlus-RIL were grown in LB medium.

Overnight grown culture of *E. coli* BL21 (DE3) and *E. coli* Rosetta gami containing the recombinant expression plasmids was diluted 1∶10 in fresh LB broth containing kanamycin and grown with vigorous shaking at 37°C for 3–4 h and when the λ_600_ nm had reached an absorbance of 0.6–0.8, a separate aliquot of uninduced culture was kept as a control. The protein expression was induced with IPTG to a final concentration of 1 mM, the temperature was decreased to 18°C and the cells were grown overnight. After 16 h, the cells were harvested at 4°C by centrifugation at 6500 *g* for 20 min. The harvested cells were resuspended in ice-cold lysis buffer (50 mM Tris/HCl, pH 7.4, 150 mM, 0.25 mg/ml lysozyme; 30 ml per litre of initial culture). The mixture was incubated for 30 min, sonicated at 4°C with 10 cycles, each cycle consisting of 30 s on and 60 s off times followed by centrifugation at 17000 *g* to separate the cell debris and the supernatant. These fractions were visualized on the SDS-PAGE and it was observed that a low quantity of the desired protein was in the supernatant and most of it was present in cell debris as inclusion bodies.

The inclusion bodies were washed three times in buffer A (50 mM Tris/HCl, pH 7.4, and 150 mM NaCl), and 0.1% Triton X-100. Later, the inclusion bodies were resuspended in buffer A containing 8 M urea and incubated for 2 h at room temperature followed by centrifugation at 20,000 *g* using a Sorvall Biofuge Stratos centrifuge (Thermo Scientific). The supernatant comprising proteins were loaded onto a cobalt affinity column equilibrated with buffer A containing 8 M urea for immobilized metal ion affinity chromatography (IMAC) purification. The column was initially washed thoroughly with buffer A containing 8 M urea and then with buffer A containing 8 M urea and 30 mM imidazole. Finally the column was eluted with buffer A containing 8 M urea and 150 mM imidazole.

The proteins were further purified through gel filtration chromatography to 99% purity using Sephadex G-50 (Sigma) XK 16/100 column in buffer (10 mM Tris, 150 mM NaCl, pH 7.0) at a flow rate of 0.5 ml/min. The fractions containing proteins were initially checked on SDS-PAGE, pooled and dialysed against 20 mM Tris/HCl, pH 8.0 and 150 mM NaCl. These purified proteins were used for all the activity studies. The proteins were quantified by Bradford method [20]. Similar procedure was followed for both Rv1430 and PE-PPE domain.

### Cloning, Expression and Purification of Recombinant Ser199Ala Mutant Rv1430 PE-PPE Domain

For site directed mutagenesis of Rv1430 PE-PPE domain, internal primers were designed and commercially obtained from Bioserve (Bioserve Biotechnologies Pvt. Ltd, Hyderabad, India). PCR amplified PE-PPE domain harboring the mutation was constructed using overlap PCR [21]. The upstream and downstream primers were 5′GGTTTC***G***CGCAGGGCGCGTCGGTCGCC3′ and 5′GCCCTGCG***C***GAAACCCAACACCACAAC3′, respectively. These are designed to replace the Ser199 with Ala and cloned under the restriction sites *Bam*HI and *Hin*dIII into pET-28a vector. The presence of mutation was confirmed by sequencing from Macrogen sequencing service (Macrogen Inc., Seoul, Korea) before transforming into the *E. coli* BL21 (DE3) CodonPlus-RIL cells. The overexpression and purification of recombinant Ser199Ala mutant PE-PPE domain was carried out as described above.

### Western Blot

The expression of the desired proteins was confirmed by Western blotting using Amersham Wet blot apparatus TE 22. Anti-His monoclonal antibody (1∶2000 dilutions) was used as primary antibody and peroxidase-conjugated goat anti-mouse IgG (1∶2000 dilutions) was used as secondary antibody. For Western blot, the protein samples were loaded and transferred to PVDF membrane (Millipore). The blot was developed with Supersignal w pico chemiluminiscent substrate (GeneX) and visualized on a versa doc V5 (Bio-Rad).

### Enzymatic Assay

The enzymatic assay of both proteins; full-length Rv1430 and its PE-PPE domain were determined using different esters of *p*-nitrophenol such as acetate (pNPC2), butyrate (pNPC4), caproate (pNPC6), caprylate (pNPC8), caprate (pNPC10), laurate (pNPC12), myristate (pNPC14) and palmitate (pNPC16). The *p*-nitrophenylesters were initially dissolved in 10 ml of 2-propanol to concentrations of 10 mM stock. The standard reaction mixture (1 ml) contained 1 mM *p*-nitrophenylester, 10 mM phosphate buffer (pH 7.0) and 30 µg (11.63 µM) of purified proteins. In the case of esters with pNPC8 and longer chain lengths, assay solution contained 10 mM sodium phosphate buffer (pH 7.0), 0.4% Triton X-100 and 0.1% gum arabic. Similar protocols were used by Schue et al., [22] and West et al., [23] in the enzymatic studies of Rv1984c, Rv3452 and a family of seven cutinase-like proteins (CULPs) in *M. tuberculosis*. The reference cuvette had all the components of the standard reaction mixture with the exception of the protein. The enzymatic hydrolysis product of all the substrates is *p*-nitrophenol and was quantified by measuring the absorbance at 410 nm using a UV-1800 Shimadzu-UV spectrophotometer. The molar extinction coefficients of *p*-nitrophenol were also determined prior to the measurements under each reaction condition. The activity was determined by measuring the initial rate of hydrolysis of various *p*-nitrophenyl esters. One unit of enzyme activity was defined as the amount of enzyme required to release 1 µmol *p*-nitrophenol/min from *p*-nitrophenylester. The activity of the Ser199Ala PE-PPE domain was tested on the most active substrate, pNPC6.

The lipolytic activity of Rv1430 and the PE-PPE domain was measured using Tween-20 (polyoxyethylene sorbitan monolaurate) as substrate as reported earlier [23], [24]. A reaction mixture was prepared with 33 mM CaCl_2_ and 0.33% Tween-20 in buffer (50 mM Tris–HCl pH 7.0 and pH 8.0; 50 mM MES for pH 6.0) in a final volume of 1 ml. 30 µg of purified protein was added to the reaction mixture for each assay at different pH and were incubated at 37°C for 1 h and the turbidity was measured at 405 nm.

The cutinase activity was measured as previously reported [25]. Apple cutin prepared from mature apples served as the substrate [23], [26]. For a typical assay, 1 mg of enzyme and 100 mg of apple cutin were added into buffer (50 mM Tris–HCl, pH 7.5), in a final volume of 10 ml. The tube was shaken at 125 rpm for 18 h at 37°C. At the end of reaction, the remaining cutin was removed by centrifugation. The resulting supernatant was acidified with acetic acid, and the released cutin monomers were extracted with chloroform. The organic soluble material was collected and dried under a stream of nitrogen. The dried cutin monomers were converted to their corresponding methyl esters and then silylated with bis-(trimethylsilyl) trifluoroacetamide (BSTFA). The silylated methyl esters were dissolved in hexane and analyzed by GC/MS QP2010 (Shimadzu, Japan) with temperature programmed as following: 125°C for 5 min, 4°C/min to 250°C, 250°C for 15 min.

### Circular Dichroism Spectroscopy Analysis of Rv1430 and its PE-PPE Domain

Circular dichroism (CD) spectra of Rv1430 (0.125 mg/ml) and its PE-PPE domain (0.15 mg/ml) were recorded at 25°C in 10 mM phosphate buffer, pH 7.0 on a Jasco J-810 spectropolarimeter, using a quartz cell with a path length of 0.1 cm. Three scans were accumulated at a scan speed of 50 nm min^−1^, with data being collected at every 1 nm from 195 to 260 nm. CDNN 2.1 [27] software was used to estimate all the secondary structural elements present in the proteins. The CD data is represented in mean ellipticity values per residue ([θ]).

### Enzyme Inhibition

The inhibitory effect of the chemical modifiers, which are specific to particular amino acids in the active site of the enzyme, such as phenylmethylsulfonyl (PMSF) to the amino acid Ser was examined. The enzyme 0.84 µM was preincubated with different concentrations of PMSF (0.5 mM to 10 mM) for 15 min at 30°C, and then the reaction was started with the addition of the most active substrate; pNPC6 and the enzyme activity was measured using the standard assay as described above.

### Effect of pH and Temperature

The enzyme activities were measured on 1 mM pNPC6 as substrate at varying pH (pH 4.0–pH 8.0) and varying temperature (25°C–45°C). The following buffers (10 mM) were used: sodium acetate (pH 4.0 and 5.0), sodium phosphate (pH 6.0 and, 7.0), Tris-HCl (pH 8.0 and 9.0) and glycine/NaOH (pH 10.0 and11.0).

### Effect of Salt

The effect of inorganic salts such as NaCl and KCl on the enzymatic activity was studied by varying the salt concentrations; 100 mM, 200 mM, 300 mM, 400 mM, 500 mM, 600 mM, 700 mM, 800 mM, 900 mM and 1 M in the assay mixture. The residual activities were measured using the standard assay with the most active molecule, pNPC6 as substrate as discussed above.

All the experiments described in this work were repeated at least in duplicate.

## Results

### Cloning, Expression and Purification of Rv1430, PE-PPE Domain and Ser199Ala Mutant PE-PPE Domain

**Figure 1:**
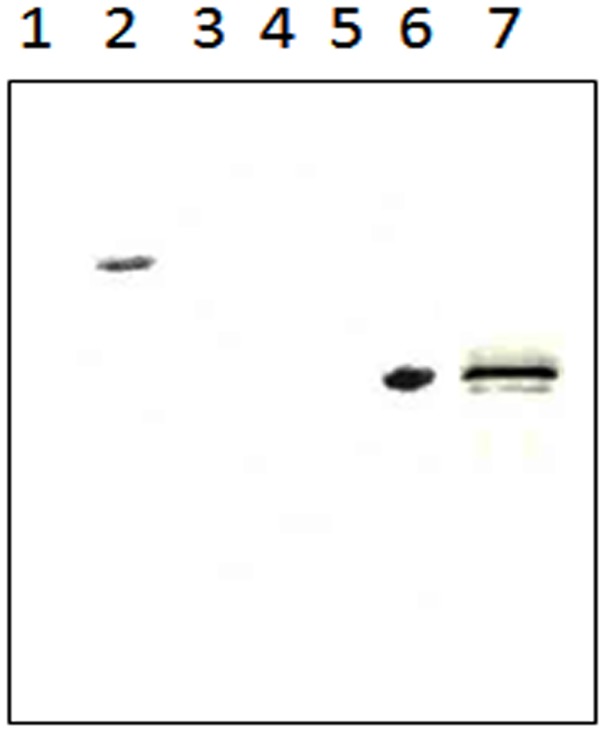
Western blot analysis of Rv1430 full-length protein and PE-PPE domain by anti-His antibody. Lane 1, Uninduced *E. coli* BL21 (DE3) CodonPlus-RIL transformed with Rv1430 construct; Lane 2, Induced sample- Rv1430 (59.3 kDa); Lane 3, Protein Marker; Lane 4, lysate of *E. coli* Rosetta gami transformed with an empty vector; Lane 5, Uninduced Rv1430 PE-PPE domain sample; Lane 6, Induced sample- Rv1430 PE-PPE domain (25.8 kDa). Lane 7, Induced sample-Ser199Ala mutated Rv1430 PE-PPE domain (25.8 kDa).

The full-length Rv1430, PE-PPE domain (144–369 amino acid residues) and mutant PE-PPE domain were cloned in pET-28a vector and the presence of the inserts was confirmed by restriction enzyme digestion. As shown in Figure S1, the DNA fragments of the desired molecular weight were observed. Further, the DNA sequencing confirmed the correct sequence of the inserts. The cloning of genes in pET-28a vector resulted in a hexa histidine tag at the N-terminus of the protein products. The proteins overexpressed by 1 mM IPTG were analysed on SDS-PAGE. The SDS-PAGE analysis showed a major protein band that corresponded to the expected molecular weight for Rv1430 and both PE-PPE domains. The expressions of the desired proteins were confirmed through the Western blot shown in Figure 1.

**Figure 2:**
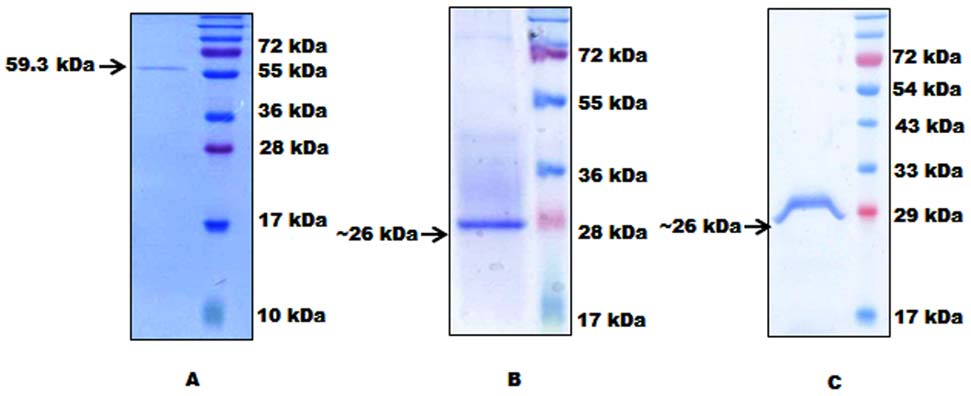
Affinity chromatographic purification of Rv1430 full-length protein, PE-PPE and Ser199Ala PE-PPE domains. Eluted fractions of the proteins recovered during the various purification steps were separated by SDS–12% polyacrylamide gel electrophoresis and stained with Coomassie brilliant blue R250. (A) Purified protein full-length Rv1430 (59.3 kDa); (B) Purified PE-PPE domain of Rv1430 (25.8 kDa); (C) Purified mutated PE-PPE domain of Rv1430 (25.8 kDa).

Under all the conditions of expression tested, the proteins were in inclusion bodies and not in the soluble fraction. Purification was therefore performed by solubilizing inclusion bodies in urea and refolding the protein by dialysis to obtain active recombinant proteins from *E. coli.* We purified the solubilized protein from supernatant using cobalt affinity chromatography. The migration of purified Rv1430 to a molecular mass of ∼59 kDa size and both PE-PPE domains to a molecular mass of ∼26 kDa from SDS-PAGE (Figure 2–A, B, C) is in agreement with the theoretical mass deduced from the DNA sequence. The purity of the proteins after gel filtration is shown in Figure S2. We obtained about 5 mg of pure protein from 1 L cell culture.

### CD Spectroscopy Data

**Figure 3:**
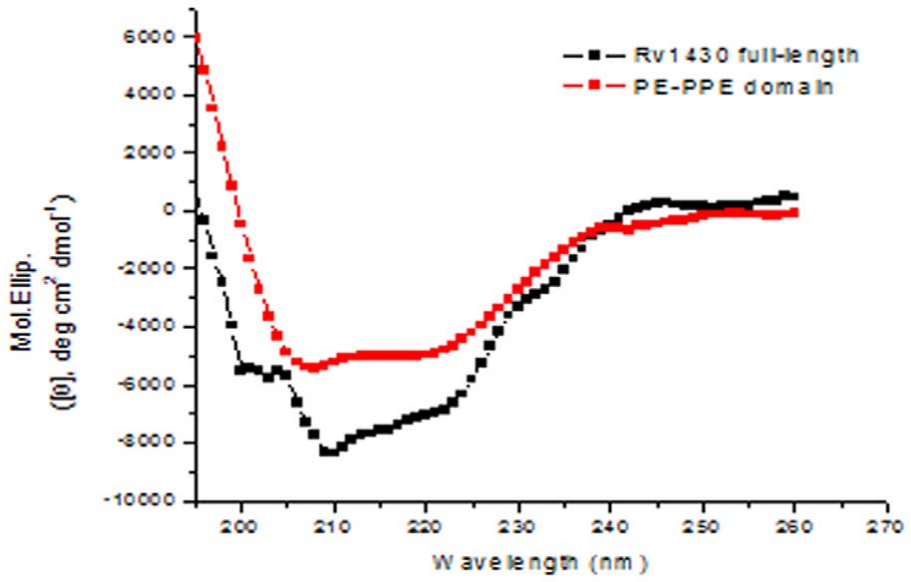
CD spectra of Rv1430 full-length (black) and its PE-PPE domain (red) measured in10 mM Tris, at pH 7.0.

To resolve if Rv1430 and its PE-PPE domain folded into native-like conformations we recorded their CD spectra and is shown in Figure 3. This result indicated that the refolded purified proteins have proper folding.

### Rv1430 and its PE-PPE Domain have Esterase Activity

**Figure 4:**
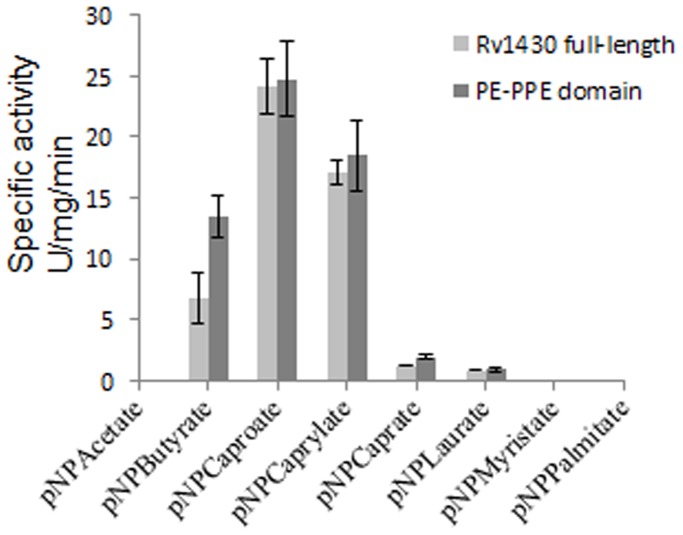
The specific enzyme activity of Rv1430 and PE-PPE domain towards the hydrolysis of various *p*-nitrophenyl ester derivatives at pH 7.0 and 37°C.

The enzymatic activity of proteins Rv1430 and its PE-PPE domain were analyzed using *p*-nitrophenyl-acetate (pNPC2), *p*-nitrophenyl-butyrate (pNPC4), *p*-nitrophenyl-caproate (pNPC6), *p*-nitrophenyl-caprylate (pNPC8), *p*-nitrophenyl-caprate (pNPC10), *p*-nitrophenyl-laurate (pNPC12), *p*-nitrophenyl-myristate (pNPC14) and *p*-nitrophenyl-palmitate (pNPC16) as substrates according to the previously reported methods [28], [29]. The enzyme activity data shown in Figure 4 indicated that at pH 7.0 and 37°C, the proteins Rv1430 and the PE-PPE domain could hydrolyze a wide range of *p*-nitrophenylester derivatives (C4–C12) substrates, of which pNPC6 was most effectively hydrolyzed. In comparison, the hydrolysis of pNPC4 and pNPC8 is lower. The activities of both proteins are quite similar as observed from this figure. From the figure it can also be seen that both proteins had no activity on pNPC2, pNPC14 and pNPC16 as substrates. The relative enzyme activity data of Rv1430 and PE-PPE domain towards the hydrolysis of various pNPs is shown in Table 1.

**Table 1:** Relative enzyme activity of Rv1430 and PE-PPE domain towards *p*-nitrophenyl derivatives at pH 7.0 and 37°C.

Substrate	Relative activity (%) of PE-PPE domain	Relative activity (%) of full-length Rv1430
*p*-nitrophenyl acetate (C2)	0	0
*p*-nitrophenyl butyrate (C4)	62	30
*p*-nitrophenyl caproate (C6)	100*	100*
*p*-nitrophenyl caprylate (C8)	79	71
*p*-nitrophenyl caprate (C10)	19	11
*p*-nitrophenyl laurate (C12)	4	2.5
*p*-nitrophenyl myristate (C14)	0	0
*p*-nitrophenyl palmitate (C16)	0	0

* The specific activity of pNPC6 is taken as 100% at pH 7 and 37°C which is ∼ 22.5 U/mg/min for both Rv1430 and PE-PPE domain.

When Tween-20 was used as substrate, no absorbance was detected at 405 nm (data not shown) indicating that Rv1430 and the PE-PPE domain do not possess lipolytic activity. When cutin, with characteristic components of C16 and C18 hydroxyl fatty acids was used as substrate, no hydrolysis products were obtained (data not shown) indicating that Rv1430 and the PE-PPE domain do not possess cutinase activity either. It was also observed that though C4 derivative of *p*-nitrophenylester was not the preferred substrate, the PE-PPE domain showed almost double the activity as compared to the Rv1430 during the hydrolysis of pNPC4 (Figure 4).

### Kinetic Properties

The kinetic constants (Km, kcat and kcat/Km) calculated from the initial rate activity of Rv1430 and its PE-PPE domain determined against active substrates pNPC4, pNPC6 and pNPC8, at pH 7.0 and 37°C are reported in Table 2. The Lineweaver–Burk plot was used for kinetic parameter analysis and the data indicated that pNPC6 is the most suitable substrate for Rv1430.

**Table 2:** The kinetic parameters of Rv1430 and PE-PPE domain.

Enzyme	Substrate	K_m_ (mM)	K_cat_ (s^−1^)	K_cat_/K_m_ (M^−1^s^−1^)
Rv1430	pNPC4	5.2	341	6.55×10^4^
	pNPC6	5.15	534	1.04×10^5^
	pNPC8	10	305	3.05×10^4^
PE-PPE domain	pNPC4	4.5	182	4.04×10^4^
	pNPC6	4.2	250	5.92×10^4^
	pNPC8	5.6	99	1.76×10^4^

### Temperature Dependent pH Tolerance and Effect of Salt Concentration on the Esterase Activity of Rv1430 and the PE-PPE Domain

**Figure 5:**
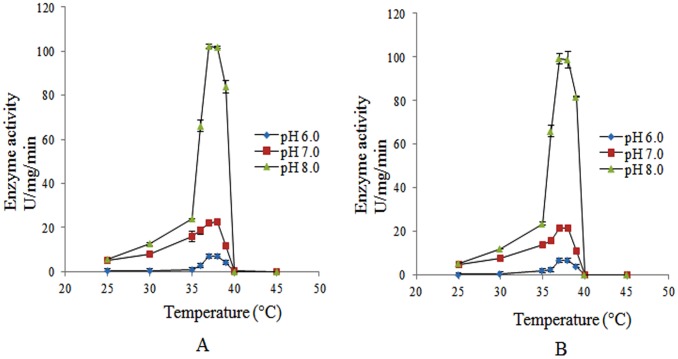
The effect of pH as a function of temperature on the enzyme activity (A) PE-PPE domain. (B) Rv1430 full-length.

The enzyme activity of the purified proteins as a function of pH within the range 4.0 to 11.0 was studied. At pH 4.0 and 5.0 no enzyme activity was detected. From pH 9.0–11.0, enzyme activity was too low to be detected since the substrates spontaneously decomposed causing a deep background. Therefore the enzyme activity of Rv1430 and its PE-PPE domain were tested at certain pH conditions (pH 6.0–8.0) in the temperature range (25°C to 45°C) using the most active substrate, pNPC6. The Figure 5-A represents the esterase activity of the PE-PPE domain as a function of pH and temperature. As shown in this figure, the highest enzyme activity was observed between 37–38°C at both pH 7.0 and pH 8.0. At 39°C, 50% inactivation and at 40°C, complete inactivation of the PE-PPE domain was observed. Similar results were also observed in the full-length Rv1430 and are shown in Figure 5-B.

**Figure 6:**
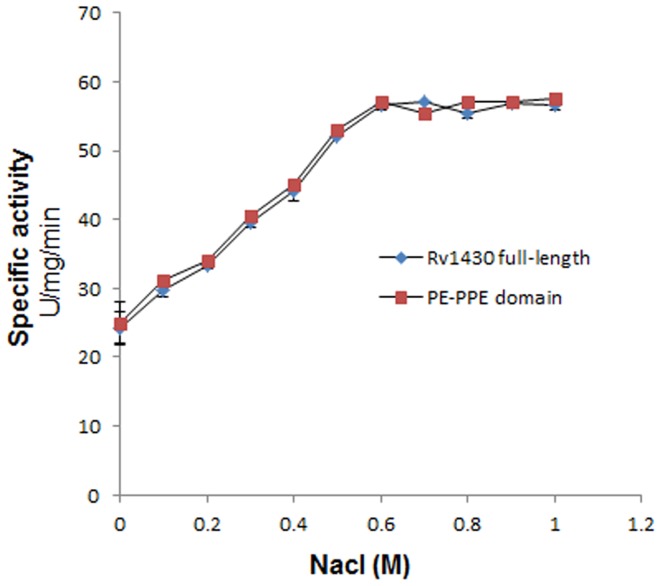
Effect of NaCl concentration on the esterase activity of Rv1430 and PE-PPE domain measured on substrate pNPC6.

To score the effect of ionic interactions on esterase activity of Rv1430 and the PE-PPE domain, the proteins were incubated with high salt in buffers, 15 min prior to the enzyme assay. It was observed that with increase in the salt concentration, the enzymatic activity of both Rv1430 and the PE-PPE domain increases. The increasing enzyme activity with increasing ionic strength is same for NaCl (Figure 6) and KCl (data not shown) and the activity increases till 600 mM and later remained constant up to 1 M.

### Rv1430 Belongs to Serine Hydrolase Family of Proteins

Earlier studies in our laboratory revealed that the Rv1430 PE-PPE domain displays a “serine α/β hydrolase” fold with a conserved Ser, Asp and His catalytic triad characteristic of lipase, esterase and cutinase activities [19]. To validate Ser199 as a part of the catalytic triad, the Ser199Ala mutant PE-PPE domain was purified and activity was measured under identical conditions to those used for PE-PPE domain. The mutant PE-PPE domain was found to be enzymatically inactive (Figure S3) on pNPC6 as substrate, which suggested that Ser199 constitutes the catalytic triad in PE-PPE domain.

**Figure 7:**
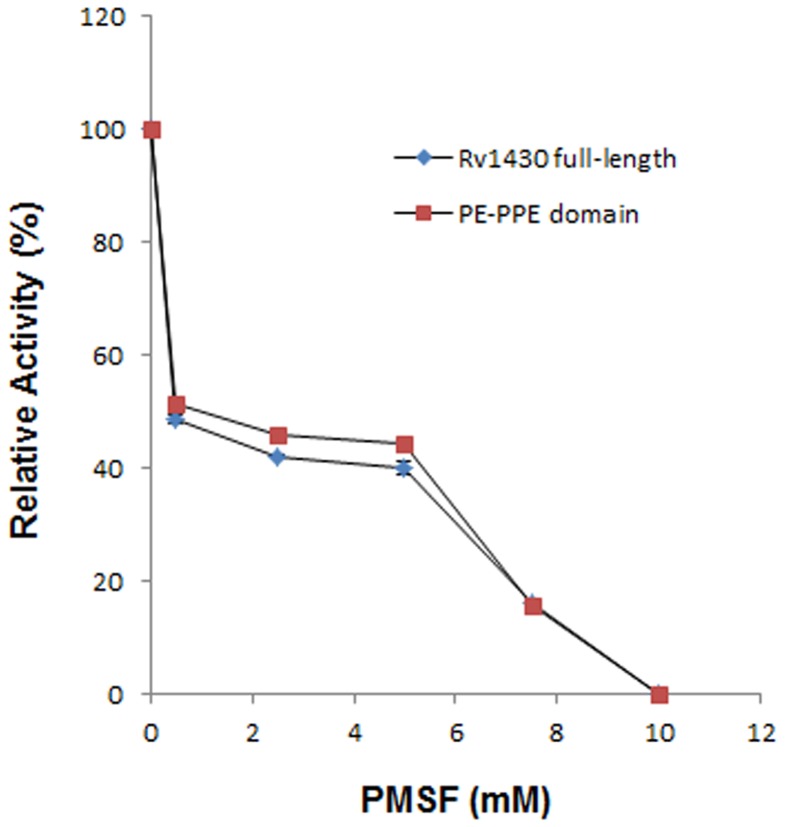
Effect of PMSF on the hydrolysis of pNPC6 by Rv1430 and PE-PPE domain.

To further confirm the role of Ser199, the enzyme was pre-incubated with different concentrations of PMSF. It is known that PMSF is a serine protease inhibitor that binds exclusively to the active site serine residue. We found that the activities of both full-length Rv1430 and the PE-PPE domain were inhibited by 50% even at low concentration of PMSF (0.5 mM). The enzyme activities were efficiently abolished at higher concentrations of PMSF when incubated for 15 min at 30°C as shown in Figure 7. This confirmed that Rv1430 is indeed a serine hydrolase.

## Discussion

The PE and PPE are two protein families of mycobacterium with limited reports on structure and function characterization. The 3D structure of PE (Rv2431c) and PPE (Rv2430c) complex was reported [30] with the PDB_ID: 2G38. The PE domain of Rv1430 shares 46% sequence homology with Rv2431c, the PDB_ID: 2G38 A chain. Our previous computational studies have shown that some PE and PPE proteins hitherto not studied comprise a conserved 225 amino acid PE-PPE domain, this domain would fold into a serine α/β hydrolase architecture and would have esterase, lipase or cutinase activity. The main aim of this work is to prove that PE-PPE domain is indeed a serine hydrolase, to find its optimal substrate, decipher the conditions for enzyme activity and stability of the protein. In order to achieve this, we cloned and purified the PE-PPE domain located at the C-terminus of Rv1430. Rv1430 is a fusion protein that comprises the N-terminal PE domain (1–87 amino acids) and a 56 amino acid linker region (88 −143 amino acids) connecting the PE and PE-PPE domains. In order to estimate the effect of these regions on the serine hydrolase activity of Rv1430, we have also cloned and purified the full-length Rv1430. To establish the activity of full-length Rv1430 and its PE-PPE domain as an esterase, the enzyme activity was studied on a series of *p*-nitrophenyl esters as substrates, Tween-20 was used as substrate to establish the activity of protein as lipase and apple cutin was used as substrate to establish the activity of protein as cutinase. The effects of pH, temperature and salt on these two purified proteins were studied to find the optimal conditions of their activity.

**Figure 8:**
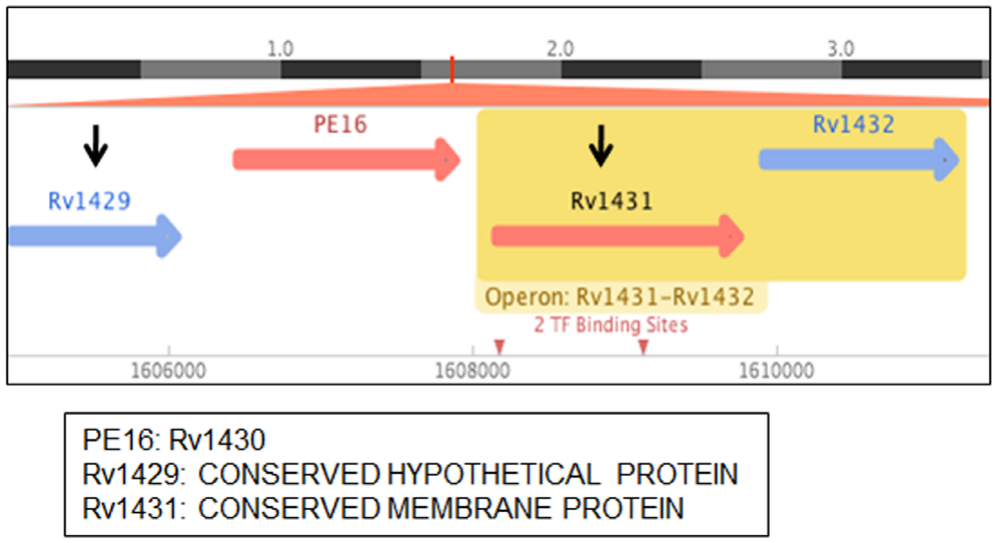
Genomic organization of Rv1430 (source: http://www.tbdb.org/and http://genolist.- **pasteur.fr/TubercuList/).**

It is well known that PE/PPE family of genes are organized in a definite pattern of operonic arrangement, where a PE is followed by a PPE gene, separated by less than 90 bp and scattered throughout the genome [13]. Some PE/PPE proteins such as Rv2430c, Rv2431c, Rv3873, Rv3812 and Rv3018c were reported to mostly form inclusion bodies when over-expressed in *E. coli*. It has been reported that PE (Rv2431c) and PPE (Rv2430c) proteins interact with and solubilize each other only when co-expressed [13], [31]–[33]. The PE16/Rv1430 is flanked by conserved hypothetical proteins and does not show any known operonic arrangement with PPE family of proteins (Figure 8, source: http://www.tbdb.org/and http://genolist.pasteur.fr/TubercuList/). The Rv1430 and its PE-PPE domain when individually overexpressed in bacterial strains were in insoluble fraction, under all the conditions of protein expressions studied by us. Hence, protein purification was initially performed under denaturing conditions which was then refolded before enzyme activity studies. The CD spectra of Rv1430 and PE-PPE domain indicated the correct folding of proteins that were refolded from the inclusion bodies.

When Rv1430 and its PE-PPE domain were tested for lipase and cutinase activities, it was observed that they could not hydrolyse Tween-20 and the apple cutin. These results indicated that the proteins do not hydrolyse higher chain length esters and that Rv1430 and the PE-PPE domain do not possess lipase and cutinase activities.

Further biochemical assays to test the activity of proteins Rv1430 and its PE-PPE domain on a wide range of substrates, esters of pNP with carbon chain lengths (C2– C16) clearly showed that Rv1430 and its PE-PPE domain is a novel esterase from *M. tuberculosis* that preferentially hydrolyzes short to medium chain (C4–C8) esters. Substrates using different esters of pNP commonly serve to differentiate lipases from esterases and maximum specific activity was observed on the substrate pNPC6. Based on these results we report that Rv1430 and its PE-PPE domain function as esterase. The mutant PE-PPE domain displayed no enzyme activity pointing to the important role of Ser199 in the enzyme activity of Rv1430. This is also supported by inhibition of activity in the presence of PMSF.

It is intriguing to observe that Rv1430 was compromised for esterase activity as compared to the PE-PPE domain with respect to C4 derivative of *p*-nitrophenylester. The potential to hydrolyse pNPC4 was almost 50% greater for the domain as compared to full-length protein (Figure 4). Based on the kinetic parameters, our studies show that the preferred substrate of Rv1430 was *p*-nitrophenyl caproate (C6), similar to that of Rv0045c, a characterized esterase of *M. tuberculosis* [34]. However, Rv1430 could catalyze C4 and C8 substrates, while Rv0045c showed better activities with C2 and C14. This indicates that mycobacterium carries an arsenal of lipase/esterases for hydrolysis of lipids of various chain lengths depending upon the availability of substrates under different conditions during in-vitro or in-vivo growth. In this context, it would be interesting to measure the expression of different estersases/lipases during different pathological conditions of mycobacterial infection.

Since the proteins are inactive at low pH and the substrates are unstable at high pH, we have used mild pH conditions (pH 6.0–8.0) to study the activity of both proteins using the most active substrate, pNPC6 over the temperature range from 25°C to 45°C. The optimal temperature for the activity of full-length Rv1430 and its PE-PPE domain was found to be 37°C and 38°C at pH 7.0 and pH 8.0. This is probably the result of *M. tuberculosis* commonly living in the bodies of humans or animals and therefore active in ambient conditions of body temperature. It was observed that the highest activity of these enzymes increased rapidly and dramatically following increased pH. Therefore, it may be hypothesized that Rv1430 like other serine hydrolases is involved in ester metabolism under less favourable conditions in this pathogen. This further supports the hypothesis that *M. tuberculosis* becomes more pathogenic at alkaline pH as proposed by [34]. From the results of the effect of salt concentration on the enzyme activity of Rv1430 and PE-PPE domain, it may be interpreted that, the secondary structure and ionic interactions related to enzyme activity of the protein is further stabilized in the presence of salt. However, higher salt concentrations (>600 mM), neither make nor break such ionic interactions.

From this work, we observed that the full-length Rv1430 and the PE-PPE domain had similar enzyme activities that are comparable. It implies that the conserved N-terminal PE domain and the C-terminal PE-PPE domain of PE16 have independent roles and that the PE-PPE domain is sufficient to exhibit the serine hydrolase activity. The presence of two independent domains in a single protein may indicate gene fusion [19], [35]. It was earlier reported that PE and PPE proteins are responsible for surface antigenic variation [1] and based on the presence of AB repeats we had shown that some PE proteins are located on the cell surface [3]. We therefore believe that the PE-PPE domain with serine hydrolase activity is located on the cell surface and one of the roles of the N-terminal PE or PPE domains would be to translocate the serine hydrolase PE-PPE domain to the cell surface site of action.

The results of inhibition studies performed in the absence and presence of PMSF, confirms that the PE-PPE domain indeed has serine esterase activity and that the protein, Rv1430 displays esterase activity.

*M. tuberculosis* is one of the oldest known human pathogens and our capability to battle against the spread of this disease continues to be inadequate and the universal health burden of this disease remains mounting [36]. In mycobacteria, lipid metabolism is thought to be one of the main pathways, since the cell wall of mycobacteria is very complex and unique among prokaryotes, it is a major determinant of virulence for the bacterium. Nearly 60% of the mycobacterial cell wall is composed of lipids. The lipid fraction of *M. tuberculosis* cell wall consists of phthiocerol dimycocerosates, mycolic acid, glycolipids, polyketides and glycans, have been found to be involved in the virulence and pathogenicity of *M. tuberculosis* [37]–[39]. Another important role of lipids in *M. tuberculosis* is that the lipids provide carbon source, promote the growth of the bacteria during the chronic infection phase [40], [41]. The required energy is stored during the long periods of bacterial dormancy which serve as a storage form of fatty acids required for membrane lipid formation [42]. This requires a variety of serine hydrolases in *M. tuberculosis* which are crucial for the survival of the bacteria and allow them to adapt to the environment provided by the host cells [43]. The success of the *M. tuberculosis* survival in host is its uniquely complex lipid rich cell wall, and cell wall synthesis pathways that are current target areas for drug development [2], [44]–[47]. Therefore, it would be interesting to see, if Rv1430, which though is considered non essential gene by Himar1-based transposon mutagenesis in H37Rv and CDC1551 strains [48], [49], when knocked out, reduces the pathogenecity of *M. tuberculosis* by making it more susceptible to antimycobacterial drugs. Thus, making Rv1430 as an attractive target for combination therapy that includes targeting the dormant mycobacteria in granulomas.

## Conclusion

The Rv1430 and its PE-PPE domain region were successfully cloned, overexpressed and the proteins were purified to homogeneity using column chromatography. The CD spectra data indicated the folded proteins after refolding and purification. Both proteins do not possess lipase and cutinase activity but hydrolyse short to medium chain fatty acid esters with the highest specific activity for pNPC6 at 37°C, 38°C and pH 7.0 and 8.0. This result is also supported by the kinetic data. These proteins are effectively inhibited by PMSF, a serine protease inhibitor and the Ser199Ala mutant PE-PPE domain had no esterase activity conforming that Rv1430 is a serine hydrolase. The esterase activity of the full-length Rv1430 and the PE-PPE domain is similar indicating that the function of the PE-PPE domain is independent of the rest of the protein.

## Supporting Information

Figure S1 Cloning of Rv1430c (1601 bp) and its PE-PPE domain (678 bp) in vector pET-28a. (A) Agarose gel electrophoresis analysis after restiction digestion of the insert Rv1430 in pET-28a (B) and Rv1430 PE-PPE domain in pET-28a. The vector and the insert bands are indicated by arrows. M: 1 kb molecular weight marker.
(TIF)

Figure S2 Silver stained SDS–12% polyacrylamide gel electrophoresis of the purified protein (A) Rv1430 full-length and (B) PE-PPE domain.
(TIF)

Figure S3 A time course of *p*-nitrophenylcaproate (pNPC6) hydrolysis at pH7.0, 37°C with PE-PPE domain (black) and Ser199Ala mutated PE-PPE domain (red) of Rv1430 protein.
(TIF)
