# Supplementary figures and images for: The PE16 (Rv1430) of Mycobacterium tuberculosis Is an Esterase Belonging to Serine Hydrolase Superfamily of Proteins
Source: PLoS One. 2013 Feb 1;8(2):e55320. doi: 10.1371/journal.pone.0055320 (PMC3562317; doi:10.1371/journal.pone.0055320)

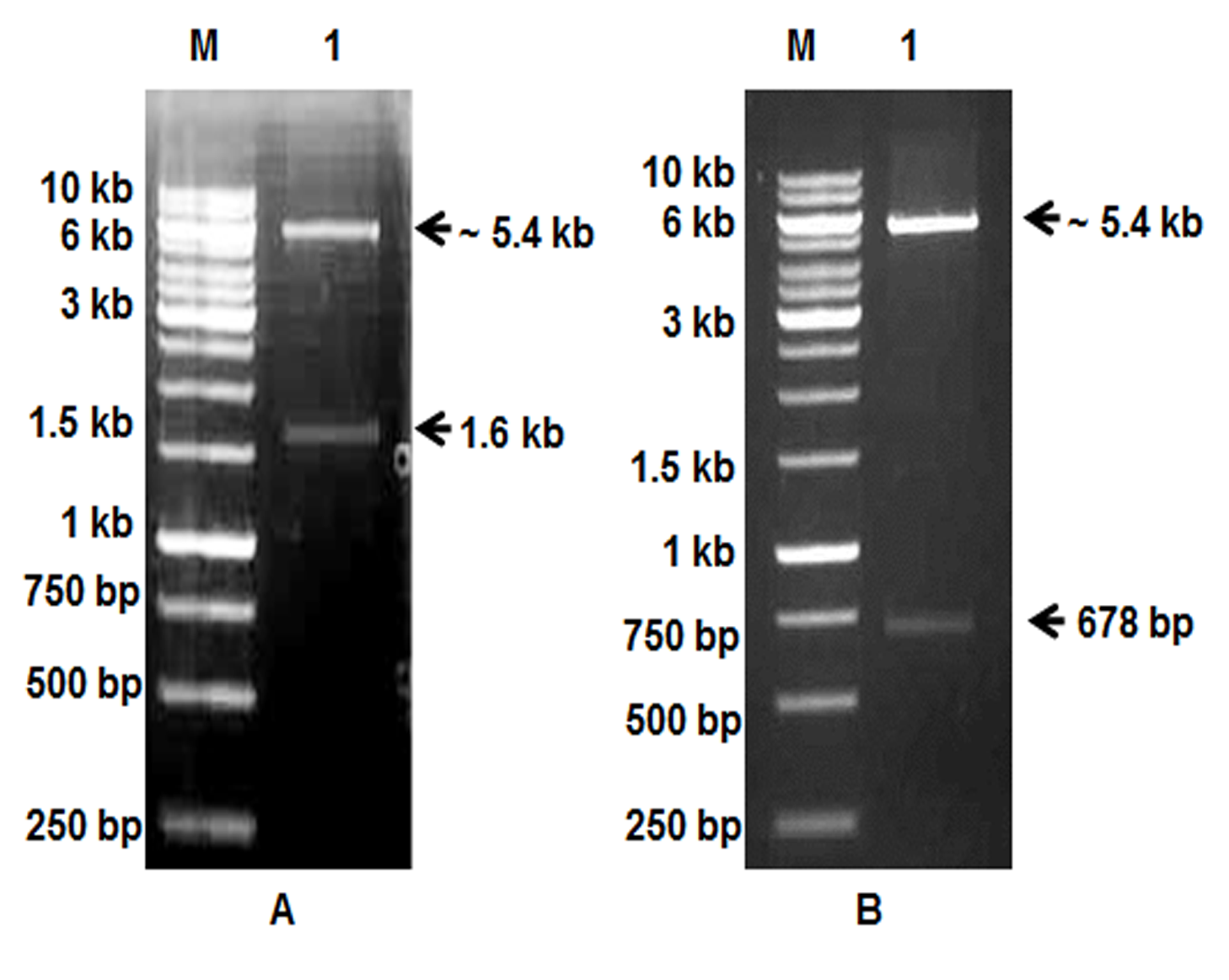

Supplement: Figure S1 — Cloning of Rv1430c (1601 bp) and its PE-PPE domain (678 bp) in vector pET-28a. (A) Agarose gel electrophoresis analysis after restiction digestion of the insert Rv1430 in pET-28a (B) and Rv1430 PE-PPE domain in pET-28a. The vector and the insert bands are indicated by arrows. M: 1 kb molecular weight marker. (TIF) [file pone.0055320.s001.tif]

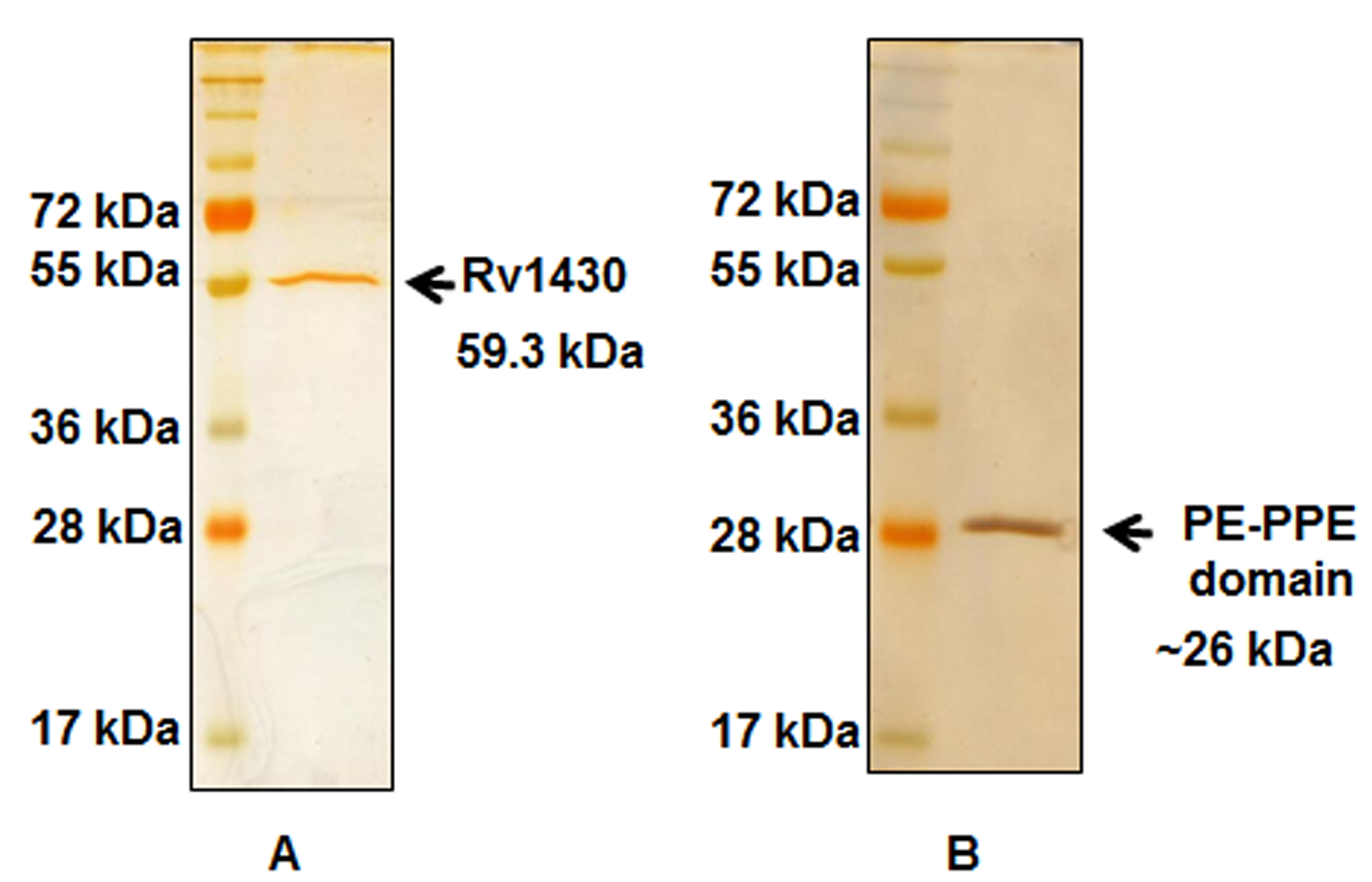

Supplement: Figure S2 — Silver stained SDS–12% polyacrylamide gel electrophoresis of the purified protein (A) Rv1430 full-length and (B) PE-PPE domain. (TIF) [file pone.0055320.s002.tif]

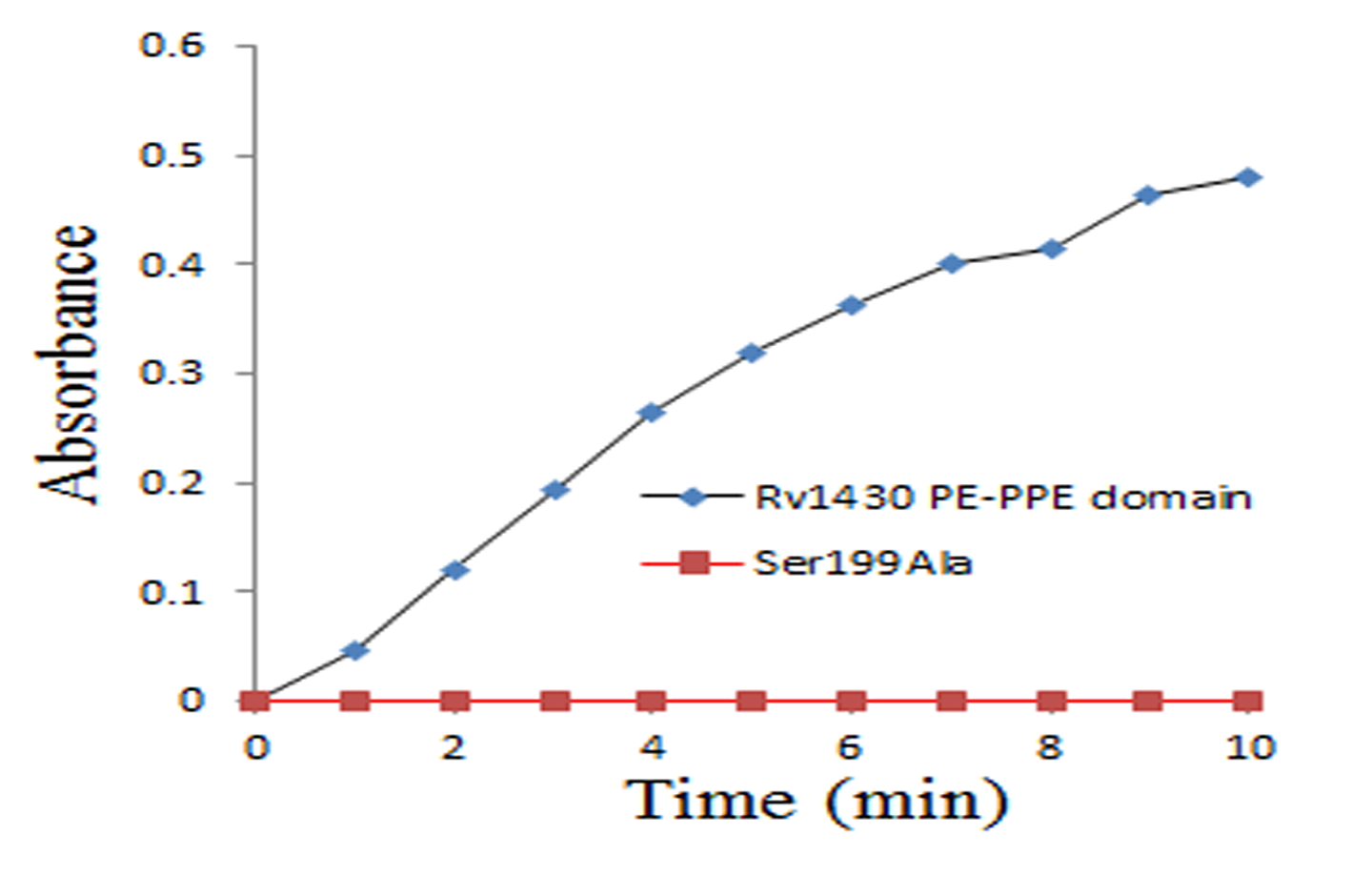

Supplement: Figure S3 — A time course of p-nitrophenylcaproate (pNPC6) hydrolysis at pH7.0, 37°C with PE-PPE domain (black) and Ser199Ala mutated PE-PPE domain (red) of Rv1430 protein. (TIF) [file pone.0055320.s003.tif]
